# Understanding the impacts of the COVID-19 pandemic on the care experiences of people with mental-physical multimorbidity: protocol for a mixed methods study

**DOI:** 10.1186/s12875-023-02106-5

**Published:** 2023-07-24

**Authors:** Matthew Menear, Arnaud Duhoux, Myreille Bédard, Jean-Sébastien Paquette, Marie Baron, Mylaine Breton, Simon Courtemanche, Savannah Dubé, Stefany Dufour, Martin Fortin, Ariane Girard, Émilie Larouche-Côté, Audrey L’Espérance, Annie LeBlanc, Marie-Eve Poitras, Sophie Rivet, Maxime Sasseville, Amélie Achim, Patrick Archambault, Virtue Bajurny, Judith Belle Brown, Jean-Daniel Carrier, Nancy Côté, Yves Couturier, Maman Joyce Dogba, Marie-Pierre Gagnon, Sergio Cortez Ghio, Emily Gard Marshall, Anita Kothari, Marie-Thérèse Lussier, Frances S. Mair, Susan Smith, Brigitte Vachon, Sabrina Wong

**Affiliations:** 1 Department of Family Medicine and Emergency Medicine, Université Laval, Quebec City, Canada; 2 VITAM Centre de recherche en santé durable, Quebec City, Canada; 3 Faculty of Nursing, Université de Montréal, Montreal, Canada; 4 Centre de Recherche Charles-Le Moyne, Montreal, Canada; 5 Person With Lived Experience (Patient Partner), Montreal, Canada; 6 Faculty of Medicine and Health Sciences, Université de Sherbrooke, Sherbrooke, Canada; 7 Person with Lived Experience (Patient Partner), Quebec City, Canada; 8 École Nationale d’administration Publique, Montreal, Canada; 9 Faculty of Nursing, Université Laval, Quebec, Canada; 10 Department of Psychiatry, Université Laval, Quebec, Canada; 11 Person with Lived Experience (Patient Partner), Toronto, Canada; 12 Department of Family Medicine, Western University, London, Canada; 13 Faculty of Social Sciences, Université Laval, Quebec, Canada; 14 School of Social Work, Université de Sherbrooke, Sherbrooke, Canada; 15 Department of Family Medicine, Dalhousie University, Halifax, Canada; 16 Department of Health Studies, Western University, London, Canada; 17 Department of Family Medicine and Emergency Medicine, Université de Montréal, Montreal, Canada; 18 School of Health and Wellbeing, University of Glasgow, Glasgow, UK; 19 Department of General Practice, Royal College of Surgeons in Ireland, Dublin, UK; 20 School of Rehabilitation, Université de Montréal, Montreal, Canada; 21 Faculty of Applied Science, University of British Colombia, Vancouver, Canada

**Keywords:** Primary care, Mental health, Multimorbidity, Chronic disease, COVID-19, Health care experiences

## Abstract

**Background:**

Primary care and other health services have been disrupted during the COVID-19 pandemic, yet the consequences of these service disruptions on patients’ care experiences remain largely unstudied. People with mental-physical multimorbidity are vulnerable to the effects of the pandemic, and to sudden service disruptions. It is thus essential to better understand how their care experiences have been impacted by the current pandemic. This study aims to improve understanding of the care experiences of people with mental-physical multimorbidity during the pandemic and identify strategies to enhance these experiences.

**Methods:**

We will conduct a mixed-methods study with multi-phase approach involving four distinct phases. Phase 1 will be a qualitative descriptive study in which we interview individuals with mental-physical multimorbidity and health professionals in order to explore the impacts of the pandemic on care experiences, as well as their perspectives on how care can be improved. The results of this phase will inform the design of study phases 2 and 3. Phase 2 will involve journey mapping exercises with a sub-group of participants with mental-physical multimorbidity to visually map out their care interactions and experiences over time and the critical moments that shaped their experiences. Phase 3 will involve an online, cross-sectional survey of care experiences administered to a larger group of people with mental disorders and/or chronic physical conditions. In phase 4, deliberative dialogues will be held with key partners to discuss and plan strategies for improving the delivery of care to people with mental-physical multimorbidity. Pre-dialogue workshops will enable us to synthesize an prepare the results from the previous three study phases.

**Discussion:**

Our study results will generate much needed evidence of the positive and negative impacts of the COVID-19 pandemic on the care experiences of people with mental-physical multimorbidity and shed light on strategies that could improve care quality and experiences.

## Background

Worldwide, healthcare systems have undergone rapid and important transformations in response to the COVID-19 pandemic. In primary care settings, clinical workspaces and workflows were re-designed to ensure the safety of patients and staff, the majority of patient care shifted to virtual care, patients and providers navigated decisions around essential vs. non-essential care, and team-based care was disrupted by staff shortages and reallocation of human resources [1–5]. Similar changes have been observed in other sectors of the health system connected to primary care, such as mental health and other specialist services [6, 7]. Concerns have been raised that these transformations are having potentially negative impacts on patients’ care experiences and health outcomes, especially those in vulnerable populations [1, 6, 8]. Among the populations likely to be most vulnerable are people living with co-existing mental disorders and chronic physical conditions, i.e. mental-physical multimorbidity. Mental-physical multimorbidity presents daily challenges and is associated with poorer outcomes than those related to mental disorders or chronic physical conditions alone, including higher levels of disability and mortality [9–11], lower quality of life [9, 12, 13], increased service use (e.g. emergency services, hospitalizations) [14–16], and higher healthcare costs [9, 17].

People living with one or more chronic conditions have also had a higher risk of acquiring COVID-19 infection in addition to severe respiratory illness and mortality [18–22]. Fear and anxiety around possible infection led some people with chronic physical conditions to avoid seeking needed medical care, increasing risk of serious illness complications [23, 24]. This situation was exacerbated by some notable disruptions to chronic disease care in primary care during the pandemic, including delayed or cancelled chronic disease assessments and consultations, reduced prevention practices, disruptions to medication renewals, and delays in access to essential medical supplies [24–28]. For people with co-existing mental disorders, the challenges of receiving adequate care may be even greater. Indeed, the pandemic has also caused widespread disruptions to mental health services, including decreased preventive services, disruptions to counselling, psychotherapy, harm reduction and emergency interventions, mental health staff shortages, and breakdowns in care continuity and comprehensiveness [29–33].

Primary care plays an essential role in promoting an integrated delivery of chronic mental and physical health care [34, 35]. That primary care and other services have been disrupted at a time when people with multimorbidity may have needed them most is a cause for great concern. To date, very few studies have explored the perspectives of health services users on their experiences in care during the pandemic, and this knowledge gap is particularly evident for people with mental-physical multimorbidity. This study thus aims to enhance understanding of how the pandemic and resulting service disruptions have influenced the care experiences of people with mental-physical multimorbidity and identify strategies for enhancing these experiences and ultimately their health and wellbeing. We expect our study to shed important light the health services impacts of the pandemic from the service users’ point of view and support ongoing and future initiatives designed maintain or improve the responsiveness and quality of care delivered to people with mental-physical multimorbidity during and beyond the current pandemic.

Our study has four specific objectives:

1. To explore the perspectives of people living with mental-physical multimorbidity on the impacts of the pandemic on their care experiences;
2. To explore the perspectives of primary care and other providers on the impacts that the pandemic has had on the care delivered to people with mental-physical multimorbidity;
3. To map the pre- and post-pandemic care journeys of people with mental-physical multimorbidity and identify factors and critical moments that had positive and negative influences on their care experiences;
4. To identify strategies for improving the care delivered to people with mental-physical multimorbidity in primary care and other settings.

## Methods

### Overarching study approach and conceptual frameworks

This is a patient-oriented research study in which patients are engaged as full partners in the study and the focus is on patient-centric priorities that aim to improve outcomes that matter to our patient partners [36]. We have notably worked with four members of our team with lived experience of mental illness and/or chronic diseases to conceive the study, define our objectives, discuss the study design, apply for and secure funding, and will continue to involve them meaningfully throughout all study phases. Our research funding includes compensation for patient partner involvement and one meeting per year will be dedicated to identifying actions to reinforce our partnership.

We will rely on two main conceptual frameworks to help guide our investigations of participants’ care experiences and journeys: the College of Family Physicians of Canada’s Patient’s Medical Home model [37] and the ‘6W’ Multidimensional Model of Care Trajectories [38]. The Patient’s Medical Home model describes the core functions of primary care that shape patients’ care experiences, including the need to provide care that is accessible, continuous, comprehensive, person-centred, team-based, and adaptive to communities. We will be mainly exploring the care experience dimensions from this model with participants. For its part, the “6W” Model of Care Trajectories reconciles different views and concepts related to trajectories into a single, integrated framework. It identifies six main dimensions of trajectories: (1) ‘Who’ – the characteristics of the patient in need of care; (2) ‘Why’ – the chronic illness trajectory; (3) ‘Which’ – the continuity between care providers in the trajectory; (4) ‘Where’ – the locations of care and care transitions; (5) ‘What’ – the service activities and treatments provided to patients; (6) ‘When’ – the types and timing of health system contacts during episodes of care. We will use these dimensions and concepts to better understand participants’ journeys and experiences through the healthcare system.

### Study design and context

**Fig. 1:**
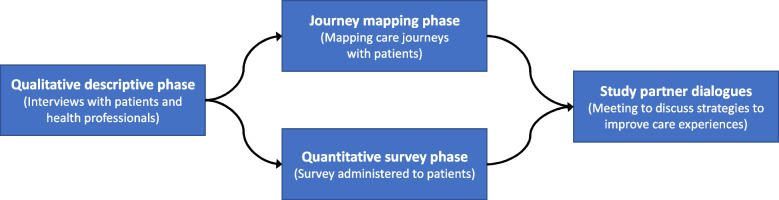
Study phases

We will conduct a mixed-methods study with multi-phase design [39] with four phases: (1) a qualitative descriptive study to explore in-depth the perspectives of people with mental-physical multimorbidity and health professionals on how the pandemic affected care experiences, as well as any ideas they may have for improvement (*objectives 1, 2 and 4*); (2) a qualitative journey mapping phase to map patients’ care journeys and the factors or key moments during those journeys that were critical to shaping their care experiences using narrative qualitative approaches alongside design thinking techniques [1–5] (*objectives 3 and 4*); (3) a quantitative survey phase to further describe the care experiences of people with mental-physical multimorbidity during the pandemic (*objectives 1 and 4*); and (4) a study partner dialogue phase to share our results with a range of actors and discuss the most promising strategies for improving the care experiences of people with mental-physical multimorbidity (*objective 4*). The initial qualitative descriptive phase will specifically inform the journey mapping and survey phases, which will be conducted concurrently, followed by the partner dialogues informed by all three previous phases. The relationships between study phases are presented in Fig. 1.

Our study is integrated within another larger study called MAVIPAN (“Ma vie et la pandémie au Québec”) [40]. MAVIPAN is a five-year longitudinal cohort study (2020–2025) launched in April 2020 that examines the psychological and social impacts of the pandemic on the Quebec population. Individuals across the province aged 14 and over were invited to complete a series of online surveys in French or English to collect data on their socio-demographic characteristics, exposure to COVID-19, mental and physical health, employment and family situation, perceived impacts of the pandemic, and other topics. Over 3000 respondents have completed the baseline survey, which was delivered via REDCAP and integrated within the PULSAR platform at Université Laval, a data sharing platform for sustainable health projects (www.pulsar.ca). However, MAVIPAN surveys have not yet examined respondents’ care experiences in a comprehensive way. The current study uses the MAVIPAN study infrastructure to facilitate participant recruitment, data collection and analysis, and the dissemination of results.

### Phase 1: qualitative descriptive phase

The first phase of this study relies on a qualitative descriptive methodology, which is well suited to helping us obtain a rich and faithful description of participants’ care experiences expressed in their own language [41]. Participants with mental-physical multimorbidity will be purposefully sampled among respondents of the MAVIPAN study. All respondents have provided data on their socio-demographic characteristics as well as their current health conditions, including any chronic mental or physical health conditions. To be eligible for this study phase, respondents will need to: (1) be aged 18 years or older, (2) have a mental disorder (self-reported using a list of 12 disorders and an open-ended question), (3) have a co-existing chronic physical condition (self-reported in questionnaires using a list of 11 conditions and an open-ended question), (4) have sought or received services for their physical or mental health needs during the pandemic, and (5) have already consented to being contacted for qualitative investigations connected with the MAVIPAN study. We will aim to maximize the diversity within the sample with respect to age, gender, region, types of conditions and health service experiences.

Healthcare professionals will also be purposefully sampled among respondents of the MAVIPAN study. Approximately one third of MAVIPAN’s participants held positions in the healthcare system as either a health professional, manager or decision maker. Health professionals (e.g., family physicians, nurses, social workers, psychologists, psychiatrists, etc.) will be eligible to participate if they provided direct care to adults with mental-physical multimorbidity during the pandemic. Again, diversity in the sample will be sought with respect to the age, gender, profession, work experience and clinical settings of participants.

The recruitment process for participants with mental-physical multimorbidity will be the same as for clinicians. From a list of MAVIPAN participants who have consented to be re-contacted for qualitative research, a list of potential participants meeting our eligibility criteria will be compiled. Email invitations to participate in the qualitative phase of our study will then be sent to them (as many as needed to ensure sufficient diversity in our sample). If individuals are interested in participating, they will be invited to contact the COVID-Care Experiences study coordinator to learn more about the study, to validate their eligibility to participate, and to schedule a date for an interview. Consent forms will be sent electronically and participants’ informed consent will be obtained and recorded orally during the scheduled virtual meeting prior to the start of the interview. Participants will receive a $50 compensation for their time. If necessary, primary care professionals will also be recruited through a practice-based research network consisting of 13 academic family medicine groups affiliated with Université Laval. An information sheet will be shared with the Research Leads for each clinic, who will then disseminate information on the project to the other clinicians at their clinic. Clinicians interested in participating will be asked to contact the study coordinator for the COVID-Care Experiences Study for more information and to schedule an interview.

The MAVIPAN survey has also collected a range of socio-demographic data from respondents that is consistent with the PROGRESS Framework (e.g., place of residence, race/ ethnicity, occupation, gender/ sex, education, socio-economic status, social capital) [42]. This data will allow us to examine and promote the diversity in our sample based on PROGRESS identity characteristics, notably on age, gender and socio-economic status.

We will conduct individual, semi-structured interviews with approximately 30–40 individuals with mental-physical multimorbidity and 10–20 health care professionals. The total number of interviews will depend on the richness of the data and our ability to reach saturation. Interviews will last approximately 90 min for people with mental-physical multimorbidity and 60 min for care providers and be conducted virtually via the platform Zoom. Interview guides will be co-designed with patient partners and informed by the Patient Medical Home model, notably by focusing on different dimensions of service quality (e.g., accessibility, continuity, comprehensiveness, patient- and family-partnered care, etc.). For people with mental-physical multimorbidity, the interviews will cover three main topics: (1) impacts of the pandemic on their health care and services, (2) their care experiences during the pandemic, and (3) their perspectives on how their care experiences could be improved. For health professionals, the interviews will also cover three main topics: (1) impacts of the pandemic on the care and services provided to people with mental-physical multimorbidity, (2) their perspectives on how the care experiences of people with mental-physical multimorbidity could be improved, and (3) aspects of care they think should be explored in the quantitative survey phase of the study. All interviews will be recorded and transcribed verbatim.

The Framework method [43] will be used to analyze data from the interviews. This method is composed of 7 stages: 1) transcription, 2) familiarisation with the interview, 3) coding (inductive and deductive), 4) developing a working analytical framework (i.e. coding hierarchy), 5) applying the analytical framework, 6) charting and summarizing the data (e.g. in a matrix or spreadsheets), and 7) interpreting the data and generating and exploring the main themes. The analysis will be conducted by at least three members of the research team and patient and clinician partners will be invited to contribute to steps 4 and 7. Transcripts will be uploaded into NVivo software to facilitate data management. Coding will be both inductive (grounded in the data) and deductive (guided by concepts in our conceptual frameworks). We will also perform analyses based on the PROGRESS Framework, which will allow us to explore how themes may differ across participants that differ in their age, gender, ethnicity, socio-economic status, etc. Team members will prepare journal notes throughout the interview and analysis processes to promote reflexivity and maintain a trace of their own thoughts, reflections and relationships with the participants and the data [44]. We will produce summaries of preliminary findings in order to discuss our results as an interdisciplinary team and determine together which dimensions and aspects of individuals’ care experiences should be prioritized for inclusion in our quantitative care experience survey. The results from service users and health professionals will be compared to establish a richer understanding of care experiences during the pandemic and to identify similarities and differences in their improvement ideas.

### Phase 2: journey mapping phase

To further deepen our understanding of how the pandemic impacted the care experiences of people with mental-physical multimorbidity, we will invite participants to engage in individual journey mapping exercises. Patient journey mapping is an emerging technique in health services research aiming to represent, from the patient’s perspective, the stages or sequence of steps they go through as they experience care for their health conditions [45–48]. The technique involves mapping participants’ interactions with the health system (e.g., consultations, treatments, transitions in care, etc.) during particular episodes of care and being attentive to the emotional experiences and critical incidents (or ‘touchpoints’) that shape their overall care experiences [45, 47]. It is an interactive, engaging and creative exercise that combines narrative storytelling and visualization to produce a holistic and user-centric graphic representation of the patient’s journey. Journey mapping techniques have been used as part of quality improvement methods for over a decade as they help make visible where sub-optimal care or poor coordination between steps in the care process may negatively influence patients’ experiences [45, 49, 50]. In the current study, we will perform a retrospective analysis of participants’ care journeys prior to and during the pandemic in order to gain a more comprehensive and dynamic picture of their overall care experiences and how these have been shaped by various factors over time.

Participants with mental-physical multimorbidity that participated in the qualitative descriptive phase of this study and that consented to remaining involved in the study will be considered for the journey mapping exercise. Specific eligibility criteria for this phase will be co-designed with our research team and patient partners, but will include considerations such as: (1) the types of health conditions that participants have and the recency of their episodes of care, (2) the types of health services they have been in contact with (for example, we may prioritize participants with clear pathways through primary care), (3) the geographic location of care, and (4) participants’ ability to accurately recollect their care experiences over the past few years (established during the initial interviews). Eligible participants that consented to this study phase will be contacted by the study coordinator to provide them with information on the journey mapping exercise and to schedule a time for this activity. Participants will receive a $50 compensation for their participation in this phase as well.

The journey mapping exercises will last approximately 90 min and take place either in-person at the VITAM research centre or virtually according to the participant’s preference. As part of our effort to create safe spaces for our participants, they will have the option of being accompanied by a family member or friend during the exercise. Participants will engage in the journey mapping exercise 3–6 months after their participation in the qualitative descriptive phase, though this timeline may be influenced by the provincial public health measures that will be in effect.

The journey mapping exercises will be informed by procedures established by previous authors [45–52]. Each exercise will begin with the patient being interviewed on his or her care journey by three members of the research team: one member that will lead the interview and two other members that will help organize the information chronologically, record it visually, and ask clarifying questions. The interviews will be semi-structured in nature and will be recorded and later transcribed verbatim. The interview guides and procedures for these interviews will be co-designed with our patient partners and informed by our conceptual frameworks and the data from the previous qualitative descriptive study phase. Participants will be asked to share details about how their mental and physical health conditions emerged and the chronological sequence of events that followed their attempts to receive care for these conditions. Schematic maps will be prepared (on large pieces of paper or virtual whiteboards) that encompass the main stages of their journey (e.g. onset of illness or new episode, seeking help, being evaluated and beginning care, receiving treatments, follow-up and recovery) and dimensions of service trajectories (based on the 6W Model). As the interview progresses, details will be added to the schematic maps and sticky notes will be used to capture and mark important events or interactions, critical decisions and incidents, emotional experiences, positive and negative outcomes, and areas and ideas for quality improvement. Particular attention will be paid to participants’ illness episodes and care experiences during the time in which their mental health problems and chronic physical conditions co-existed. The research team and participants will re-arrange sticky notes and collaboratively map the patient journey until a final draft version of the map that satisfies the participant is produced. After the activity, the interviewers will work to transform the sticky pads into several visual representations (capturing service patterns and critical moments) that will be shared with the participant for their validation.

Interview and visual data from the journey mapping exercises will be analyzed using Framework and content analysis approaches, respectively. The Framework approach (described above) will be used to code and interpret data within the interview transcripts. Members of the research team involved in the interviews will carry out the analysis, informed by the constructs within our conceptual frameworks. This analysis will help produce summary sheets that will supplement each visual journey map with more information about the patient and their care experiences before and during the pandemic. It will also help us identify common or contrasting themes or sub-themes across participants involved in the mapping exercise as well as a list of ideas for quality improvement generated by participants. The analysis of interview data will be supported by NVivo software.

Content analysis, an approach commonly used to analyze visual data or materials [53], will be used to explore the similarities and differences across participants’ visual journey maps. Content analysis aims to describe and quantify phenomena and involves the flexible creation of codes and categories according to inductive and deductive coding approaches. This analysis will allow us to compare participants’ experiences at each stage of the service trajectory and examine patterns in events, critical moments, emotional responses, and outcomes. Using the data from individual journeys, we will prepare one or multiple generic journey maps that encapsulate in a visual format the most common challenges and critical incidents experienced by people with multimorbidity, accompanied by participants’ recommendations on how these challenges could be addressed. We will also attempt to compare and contrast patient journey maps according to gender and other PROGRESS characteristics (e.g. socio-demographic status) in order to further explore inequities in care experiences.

### Phase 3: quantitative survey phase

In phase 3, we will perform a cross-sectional survey of the care experiences of respondents participating in the longitudinal MAVIPAN study. This survey will be informed by the qualitative descriptive study phase and co-designed with patient partners in order to quantitatively assess care experiences and their determinants from a broader sample of participants. Respondents that completed MAVIPAN’s baseline (T0) survey (*N* = 3189) will be invited to participate in the follow-up survey and complete questions related to their care experiences during the pandemic. For the purposes of this study, we will only analyze the data of adult respondents (18 years and older).

MAVIPAN’s baseline survey collected data on respondents’ socio-demographic characteristics, exposure to COVID-19, mental and physical health, employment and family situations, and perceived impacts of the pandemic. Follow-up surveys collect similar information but can also collect additional data on specific topics aligned with the MAVIPAN study objectives. As part of our follow-up survey, we will collect new data on respondents’ care experiences since the start of the pandemic. Consistent with our Patient Medical Home framework and best practices in this area [54], we will examine several dimensions of the care experience, including access to care, exposure to team-based care, comprehensiveness, continuity of care, and person-centeredness. Survey items will be drawn from validated tools such as the Patient Perception of Patient-Centeredness Questionnaire [55] and from previous patient experience surveys conducted in Quebec [56–58] and Canada [59–62]. The specific items used will be determined based on our analyses of data from the quantitative descriptive phase and on the feedback received from patient partners and other members of our interdisciplinary team. If necessary, new items may be developed with our patient and knowledge user partners to better assess pandemic-specific impacts on care experiences. In addition, the Perceived Need for Care questionnaire [63] will be used to assess met and unmet needs for care. The survey will be pilot tested by our patient partners prior to launch.

We will conduct exploratory analyses in using descriptive statistics to characterize all variables and bivariate and multivariate regression analyses to examine associations between our variables of interest. We will group participants by their multimorbidity profile, comparing the care experiences of respondents with mental-physical multimorbidity to two other groups of respondents: (a) respondents with chronic physical conditions only, and (b) respondents with mental health conditions only. We will use a series of regression analyses to specifically examine the influence of mental-physical multimorbidity (independent variable) on our variables related to experiences of care, while accounting for the influence of various confounding variables (e.g. age, gender, socio-economic status, etc.). Gender-based analyses will be conducted in which we stratify our survey results by gender groups and include gender as a key variable in our regression analyses. Data analyses will be conducted by a MAVIPAN biostatistician using R software.

### Phase 4: study partner dialogues

The final phase of the study will involve three meetings with key study partners including patients, clinicians, managers and policymakers in order to share the results of the study and deliberate about the strategies and opportunities for improving the care experiences of people with mental-physical multimorbidity. These meetings will take the form of “deliberative dialogues”, which are a knowledge translation strategy that brings various health system actors together to learn from one another and from the research evidence, and share their views about a specific problem, options for addressing it and implementation considerations [64, 65]. The deliberative nature of these dialogues promotes structured conversations that value listening as much as speaking, the sharing of diverse perspectives, informed and reasoned debate, and the development of collaborative understanding and trust among participants [66]. Deliberative dialogues are a promising strategy to advance the use of evidence in service planning and policy [65].

We expect approximately 20–25 people to attend each of the dialogues, including primary care and mental health providers, health managers from multiple health regions, policymakers from the Quebec ministry of health, and representatives from provincial patient associations or research networks (e.g., Diabetes Action Canada, Réseau-1 Québec). Members of the research team, including patient partners, will also be invited to attend. The dialogues will each last 3 h and take place in person (if permitted by public health measures), with virtual participation also a possibility for some participants. The meeting(s) will be facilitated by the principal investigator and will be audio recorded. Documentation (e.g., brief summaries of study findings, generic journey maps, list of quality improvement ideas, etc.) will be sent to participants prior to each dialogue.

Each dialogue will focus on a particular topic. Dialogue 1 will involve a presentation by the research team of the main findings of the study regarding the care experiences of people with mental-physical multimorbidity prior to and during the pandemic. Preparation for this meeting will include pre-dialogue workshops in which the research team synthesizes evidence from across all three study phases to draw out the most salient and actionable findings to share with participants. These results will be presented during the dialogue and discussed and placed in context by dialogue participants. Dialogue 2 will involve a discussion surrounding the ideas and opportunities to adapt or improve services for people with mental-physical multimorbidity, drawing once again from the results of all study phases and the perspectives of study participants and all study partners. Dialogue 3 will focus on the anticipated challenges of implementing these service improvements and strategies needed to overcome these challenges. The audio recordings of the dialogue(s) will be transcribed verbatim and a brief summary of the meeting(s) will be shared with participants. Data from the stakeholder dialogues will be analyzed using a conventional qualitative content analysis approach [53]. Coding will be undertaken by members of the research team with involvement of patient partners. A preliminary report presenting the main findings of each dialogue will be prepared and circulated among the research team to solicit feedback and help prepare a final version.

## Discussion

In healthcare settings, multimorbidity is often the norm and not the exception. Over 50% of adult primary care patients in Canada have two or more chronic diseases, with mental health conditions being one of the most common types of co-occurring conditions [67]. People with multimorbidity, and especially those with mental-physical multimorbidity, depend on a healthcare system that can manage their multiple health needs in a responsive and integrated way. However, it remains unclear whether the transformations and disruptions that occurred in primary care and other sectors resulted in important negative effects (or even positive effects) on the quality of their care. The proposed study will thus address these knowledge gaps and generate new knowledge on the care experiences of people with mental-physical multimorbidity during this global pandemic. The study will provide clinicians, decision-makers and policymakers with helpful information about these individuals’ positive and negative experiences in care, which will guide their current and future efforts to improve service quality. Among the strengths of the study are our patient-oriented approach and our use of multiple methodological approaches to shed light on the experiences and perspectives of people with mental-physical multimorbidity. Our study partner dialogues will also represent an important step in the process of translating our evidence into concrete strategies that will have the potential to address service users’ current service needs and improve their health and well-being.

While our study has several strengths, it also has some potential limitations. The non-probabilistic sampling approach used for the larger MAVIPAN study may introduce some sampling error into our study, and result in either a lack of or over-representation of population groups that could affect both the qualitative and quantitative phases. Furthermore, our participants will mostly be drawn from the larger MAVIPAN study, which itself relies on online surveys as its principal means of data collection. As a result, our participant samples may skew towards people with greater Internet access and potentially omit people with multimorbidity with more limited access to Internet or lower digital literacy. Finally, we acknowledge that the journey mapping phase will be a challenge given that our sample of participants in the qualitative descriptive phase will be heterogeneous in terms of their socio-demographic and illness characteristics. It may thus be challenging to synthesize results across participants and create generic maps if their care journeys are too dissimilar. If this were to be the case, we would focus on summarizing the patterns and common and divergent findings that were observed across the participants in this study phase.

Despite the potential limitations, this study has the potential to generate new and important insights into the impact of the pandemic on patient care experiences – a central component of the quadruple aim [68] – among a vulnerable population group living with mental-physical multimorbidity.

## Data Availability

The MAVIPAN Database will be shared in accordance with the Canadian Institute of Health Research (CIHR) joint statement on sharing research data, the FAIR Guiding Principles for Scientific Data Management and Stewardship, and the norms established by the Ethic Committee of record. The specific datasets used and/or analyzed for the current study will be available from the corresponding author on reasonable request.
